# A Case of Yellow Nail Syndrome Complicated with Pulmonary Infection Due to *Nocardia cyriacigeorgica*

**DOI:** 10.3390/idr16050072

**Published:** 2024-09-18

**Authors:** Qiuyu Li, Jiajia Zheng, Qiuyue Zhang, Ying Liang, Hong Zhu, Yongchang Sun

**Affiliations:** 1 Department of Respiratory and Critical Care Medicine, Peking University Third Hospital, Beijing 100191, China; liqiuyu00@bjmu.edu.cn (Q.L.); zhuhong_bysy@163.com (H.Z.); suny@bjmu.edu.cn (Y.S.); 2 Department of Laboratory Medicine, Peking University Third Hospital, Beijing 100191, China; zhengjiajia@bjmu.edu.cn; 3 Department of Lymphatic Surgery, Beijing Shijitan Hospital Affiliated to Capital Medical University, Beijing 100038, China; zhangqiu3660@gmail.com

**Keywords:** yellow nail syndrome, bronchiectasis, *Nocardia cyriacigeorgica*, case report

## Abstract

Yellow nail syndrome (YNS) is a rare clinical syndrome characterized by nail bed changes, pulmonary involvement, and lymphatic drainage disorders. Pulmonary involvement usually manifests as bronchiectasis, bronchiolitis, and pleural effusion. There are few studies on yellow nail syndrome combined with opportunistic infection. Here, we report a case of clinically diagnosed YNS combined with *Nocardia cyriacigeorgica* infection and the course of treatment used, which can provide some useful information for clinicians to better understand this rare illness.

## 1. Case Presentation

**Figure 1:**
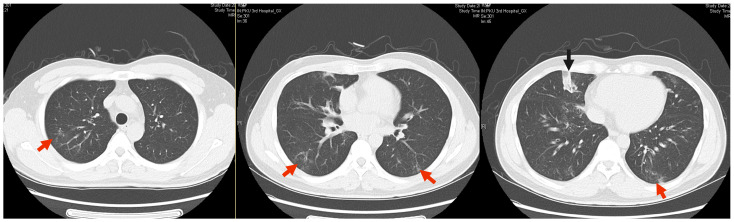
Multiple lesions of tree-in-bud (indicating bronchiolitis) were observed around the subpleural region of both lungs (red arrows), along with focal consolidation (black arrow) in the right middle lobe (10 February 2017).

**Figure 2:**
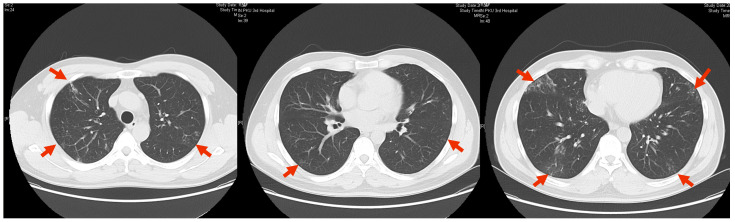
The lesions of tree-in-bud (indicating bronchiolitis) around the subpleural region of both lungs (red arrows) were increased (20 January 2022).

A 40-year-old male patient was admitted to our hospital on 26 January 2022 with a chief complaint of “recurrent cough and expectoration for eleven years, with exacerbation over the past two months”. Eleven years ago, he developed a cough accompanied by yellow purulent sputum. There were no symptoms of fever, dyspnea, or chills. He sought medical attention at another hospital where a chest computer tomography (CT) scan revealed “multiple centrilobular nodules and tree-in-bud opacities in the lungs, along with a few subpleural consolidations and thickening of bronchial walls” (Figure 1). The lesions partially resolved after oral administration of moxifloxacin 0.4 g once daily. Over the ensuing years, his symptoms and pulmonary lesions observed on chest CT scans had repeatedly worsened, yet they responded to various antibiotic treatments. During this period, an array of examinations for screening tuberculosis was conducted, including a PPD test (+++) and T-SPOT.TB test (−). Four years ago, he sought further evaluation for pulmonary tuberculosis at another hospital, where a bronchoscopy was performed. The acid-fast staining of the smear was negative. Neither mycobacterium *tuberculosis* nor *nontuberculous mycobacterium complex* was detected in his bronchoalveolar lavage fluid. Two months ago, his condition gradually deteriorated, accompanied by cough and increased expectoration along with intermittent low fever. A CT scan showed patchy ground-glass opacities, localized bronchiectasis, and consolidations in both lungs, which were significantly more extensive than before (Figure 2).

Medical history: The patient had chronic sinusitis for 2 years. His fingers and toenails were of slow growth and deformed, accompanied by color alterations and wrinkle-like changes on the surface of the nail bed in the past year. He had been smoking for 3–4 years and had given up for 6 years. He drank occasionally. Familial hereditary diseases were denied.

**Figure 3:**
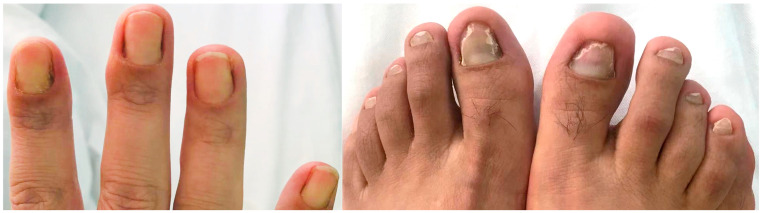
Multiple nails on both hands exhibited atrophy, with dark yellow nail plates and uneven surfaces. Additionally, there was thickening of the distal toenails on both feet, along with visible rough yellow layers.

Physical examination: The patient looked well and had normal body temperature upon admission. No rash or subcutaneous nodules were found on his body. No superficial lymph nodes were palpable. The respiratory sounds of both lungs were clear, without rhonchi or rales. The heart rate was 68 beats/min, with a regular rhythm. Multiple nails of both hands were atrophic, with dark yellow nail beds and uneven surfaces. The distal ends of the toenails of both feet were thickened and rough, with visible yellow alteration (Figure 3).

**Figure 4:**
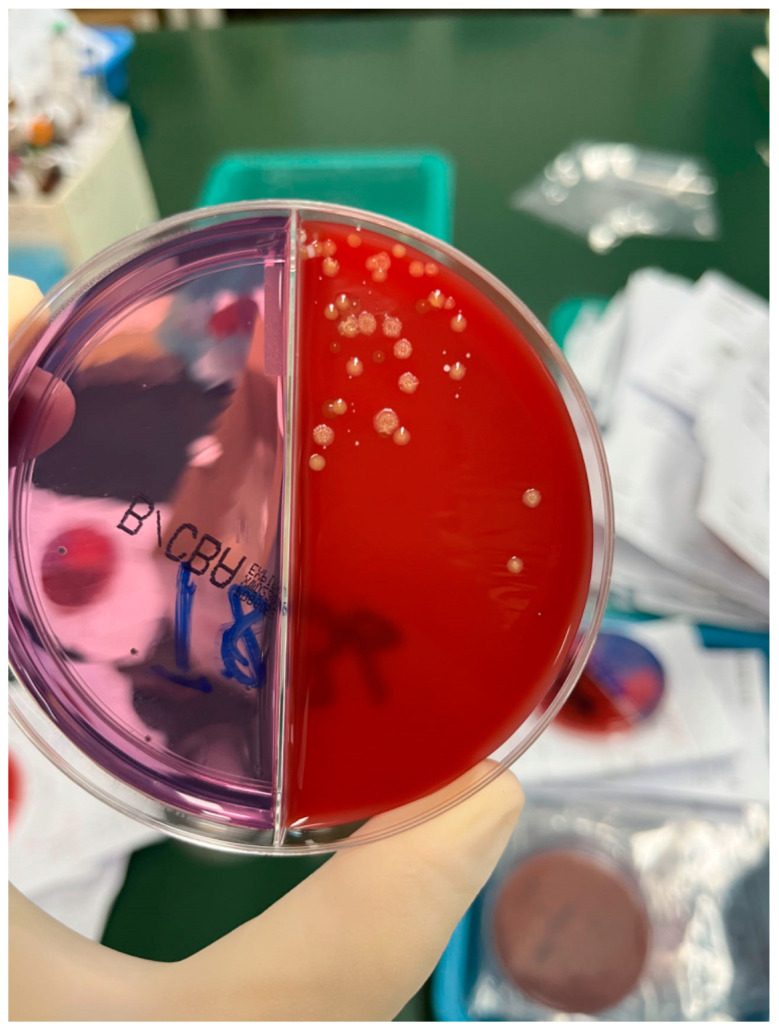
The colony morphology of Nocardia cyriacigeorgica in this patient. The colonies exhibited a dry, white appearance with wrinkled surfaces, manifesting a characteristic “biting agar” phenomenon.

**Figure 5:**
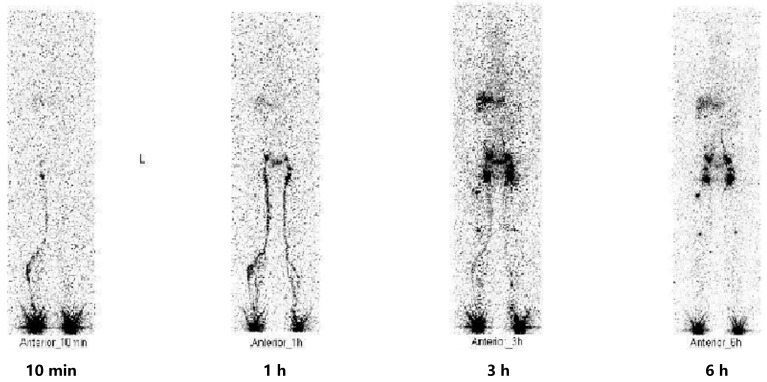
Lymphangiography in both lower limbs: Injection of 99mTc-DX; injection dose: 2 × 5 mCi; subcutaneous injection of imaging agent between the first, second, fourth, and fifth toes of both feet, 10 min, 1, 3, and 6 h for whole-body imaging. Lymphatic vessels of bilateral legs could be clearly displayed. Delayed lymphatic drainage in both lower limbs was present.

**Figure 6:**
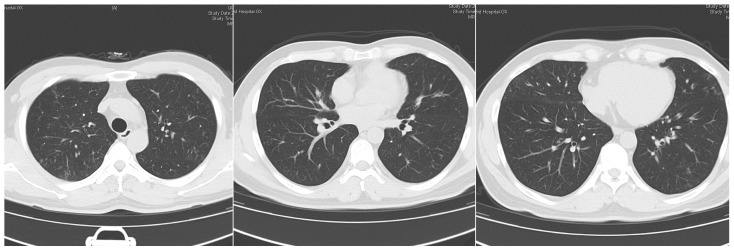
After treatment, the lesions in both lungs were improved (11 March 2022).

**Figure 7:**
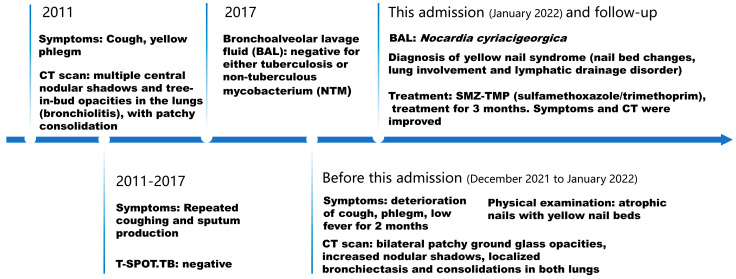
The timeline for this case.

The patient’s routine laboratory examinations were largely normal. During his hospitalization from January to February 2022, T-SPOT.TB, 1,3-β-D Glucan (G), and Galactomannan (GM) tests were negative. All antibodies associated with autoimmune diseases were also negative. Total IgE was slightly elevated, while *Aspergillus fumigatus*-specific IgE was negative. Pulmonary function testing revealed obstructive ventilatory dysfunction (FEV_1_/FVC 66%, FEV_1_%pred 69%) with a mild reduction in diffusing capacity. Bronchoalveolar lavage showed a total cell count of 12 × 10^6^/L, comprising 4.5% macrophages, 87% lymphocytes, and 8.5% neutrophils. Bronchoalveolar lavage fluid (BALF) was subjected to comprehensive pathogen detection and antimicrobial susceptibility testing. The specimen underwent Gram staining, revealing Gram-positive, right-angled branched, and cotton-like mycelia under microscopic examination, alongside a positive result for weak acid-fast staining. Following an 18–24 h incubation period, pinpoint-sized colonies emerged on the blood agar plate. These colonies grew at a slow pace, gradually expanding in size after 48 h of subculturing. The colonies exhibited a dry, white appearance with wrinkled surfaces, manifesting a characteristic “biting agar” phenomenon (Figure 4). Matrix-assisted laser desorption/ionization-time of flight mass spectrometry (MAL-DI-TOF MS) definitively identified the colonies as *Nocardia cyriacigeorgica*, with a comparison score exceeding 1.5 in the top ten results (Table 1). Antimicrobial susceptibility testing was conducted using the broth microdilution method to determine the minimum inhibitory concentrations (MICs) for a panel of antibiotics. Additionally, next-generation sequencing (NGS) metagenomic analysis was performed to detect the potential pathogens in the bronchoalveolar lavage fluid. In this patient, there were 11 sequences of *Nocardia cyriacigeorgica* genes detected, which were confirmed by submitting these sequences to the BLAST database (https://blast.ncbi.nlm.nih.gov/, accessed on 25 January 2022) (Table 2). Therefore, *Nocardia cyriacigeorgica* infection was identified in this patient. Drug sensitivity testing indicated that this strain was susceptible to ceftriaxone, cefixime, imipenem, sulfamethoxazole/trimethoprim, tobramycin, and amikacin. Lymphatic radionuclide imaging demonstrated delayed lymphatic drainage in both lower limbs, suggesting a disorder of lymphatic flow (Figure 5). Based on the bilateral bronchiolitis, localized bronchiectasis, changes in nail bed color and shape, and the lymphatic drainage disorder, the patient was clinically diagnosed with “yellow nail syndrome (YNS)”, complicated by *Nocardia cyriacigeorgica* infection. Oral sulfamethoxazole/trimethoprim 1.6 g/0.32 g twice a day was initiated. Due to renal insufficiency during the treatment, the regimen was adjusted to sulfamethoxazole/trimethoprim 0.4 g/0.08 g twice daily combined with minocycline 0.1 g twice daily. After three months, his respiratory symptoms improved, and the lesions on chest CT partly resolved (Figure 6), with renal function normalized. The sputum specimen from the patient exhibited a negative bacterial culture result, and there were no instances of recurrent exacerbation during the follow-up period. The timeline for this case is shown in Figure 7.

## 2. Discussion

In our case, the patient exhibited the following clinical features: (1) recurrent bronchiolitis and localized bronchiectasis; (2) suspected “yellow nail” changes, including nail bed deformity, color alteration, multiple wrinkle-like surface changes, and slow nail growth; (3) evidence of lymphatic drainage disorder in both lower limbs; and (4) absence of other etiologies of bronchiolitis or bronchiectasis, including tuberculosis, nontuberculous mycobacteria (NTM) or fungal infections, connective tissue diseases, immunodeficiency disorders, and congenital abnormalities. Consequently, he was clinically diagnosed with yellow nail syndrome (YNS).

YNS is a rare clinical syndrome characterized by the classic triad of yellow nails, lymphedema, and respiratory system diseases. Diffuse bronchiectasis is a common clinical manifestation of respiratory involvement. The term “Yellow nails” was first described by Heller in 1927. In 1964, Samman and White reported a series of cases of nail color changes, slow growth, and ankle edema, probably due to lymphatic drainage impairment, and they proposed the concept of “yellow nail syndrome” [1]. Subsequently, Emerson found in 1966 that patients with YNS were often accompanied by pleural effusion [2]. In 1979, Runyon et al. proposed the classical triad of YNS: yellow nails, lymphedema, and pleural effusion [3]. Valdes et al. and Woodfield et al. analyzed patients with YNS who had pleural effusion and diffuse bronchiectasis, finding that 14% to 46% of YNS patients had pleural effusion, and half had diffuse (or multiple) bronchiectases, commonly affecting the lower lobes [4,5]. The pathogens in these patients during respiratory infections are similar to those in idiopathic bronchiectasis patients, often with *Pseudomonas aeruginosa* colonization in the airways. However, the prognosis of YNS with bronchiectasis is usually better than idiopathic diffuse bronchiectasis, although mucous plugs are more common in YNS. A diagnosis of YNS can be made with the presence of two out of the three criteria: “yellow nails”, respiratory system involvement, including bronchiolitis, bronchiectasis, and/or pleural effusion, and lymphedema. Yet, Hiller et al. proposed that “yellow nails” should be an essential condition, with an incidence of 85% to 100%, respiratory involvement should have an incidence ranging from 39% to 100%, and lymphedema should have an incidence from 29% to 80%, with the complete triad present in 27% to 76% of cases [6]. Currently, there is still no definitive treatment recommendation for this syndrome. Generally, treatments are tailored to different clinical presentations, and yellow nails in some patients may spontaneously resolve or improve with the overall condition. Treatments for YNS patients with bronchiectasis are similar to those for idiopathic bronchiectasis, such as vaccination to avoid respiratory infections, enhanced postural drainage and sputum removal, and utilizing sensitive antimicrobial therapy for respiratory infections.

The detection of *Nocardia cyriacigeorgica* was another feature in our case that had not been described in previous case reports. Pulmonary nocardiosis, an opportunistic infectious disease caused by *Nocardia cyriacigeorgica*, typically occurs in immunocompromised patients but can also affect immunocompetent individuals, particularly those with pre-existing lung diseases. Risk factors for pulmonary nocardiosis include corticosteroid therapy, chronic obstructive pulmonary disease (COPD), cystic fibrosis, and bronchiectasis [7,8]. In recent years, there have been limited reports of pulmonary bronchiectasis complicated by Nocardia infection [9,10]. In these cases, while the overall immune status was normal, local cellular immunity was compromised due to bronchiectasis. The presence of *Nocardia cyriacigeorgica* in lower respiratory tract specimens should not be overlooked or merely considered colonization, especially in patients with chronic respiratory diseases. For patients with severe infections, empirical treatment with a combination of two or three antibiotics is recommended. For patients with mild to moderate cases of pulmonary nocardiosis and uncomplicated skin infections without systemic involvement, monotherapy may be sufficient. According to a US multicenter survey, TMP-SMX is used as the first-line treatment of *Nocardia cyriacigeorgica* [11]. If the patient is allergic to sulfonamides, desensitization should be carried out as far as possible. If the patient is not suitable for desensitization or cannot tolerate sulfonamides, alternative drugs should be selected based on the drug sensitivity test results. In vitro drug sensitivity tests and animal models of disease have confirmed that a variety of antibiotics have anti-Nocardia activities, including amikacin, imipenem, meropenem, third-generation cephalosporins (ceftriaxone and cefotaxime), minocycline, extended-spectrum fluoroquinolones (such as moxifloxacin), linezolid, and tigecycline. Some case reports showed that these drugs can successfully treat patients with nocardiosis [11,12,13]. In our case, as the patient had renal insufficiency due to oral sulfamethoxazole/trimethoprim, the dose of sulfamethoxazole/trimethoprim had to be reduced and combined with minocycline. After long-term therapy, his condition improved, and the lesions in his lungs resolved gradually.

## 3. Conclusions

In summary, YNS is a rare clinical syndrome with an unknown etiology. Given its association with bronchiectasis, a thorough medical history and physical examination are crucial for diagnosis. Lymphatic imaging can be instrumental in confirming suspected cases. Furthermore, YNS complicated by Nocardia cyriacigeorgica infection is exceptionally rare, with airway structure damage attributed to YNS identified as a significant risk factor for pulmonary nocardiosis, which should not be overlooked in individuals with chronic respiratory diseases.

## Figures and Tables

**Table 1:** MAL-DI-TOF MS analysis of cultured colonies.

Detected Species	Log (Score)
Nocardia cyriacigeorgica NO 23 HUA	1.602
Nocardia cyriacigeorgica 121106 01 HUA	1.557
Nocardia sp. N1064 IBS	1.554
Nocardia cyriacigeorgica 120619 13 HUA	1.543
Nocardia cyriacigeorgica 120619 20 HUA	1.487
Nocardia sp. N394 IBS	1.447
Nocardia cyriacigeorgica 121106 16 HUA	1.443
Nocardia cyriacigeorgica 120619 18 HUA	1.435
Nocardia cyriacigeorgica NO 24 HUA	1.432
Nocardia cyriacigeorgica 120619 08 HUA	1.418

**Table 2:** Sequences of genes submitted to BLAST database for species identification.

Sequences	Description	Total Score
GCCGCCCGAATCGGGCTGATCACCGAGATCAGCGCCGATCCGATGGCCGA	Nocardia cyriacigeorgica strain 3012STDY6756504 genome assembly, chromosome: 1	87.9
ATGCAGGTAGCCGAGATGACGTTCGTATTGGTCGAGGATGTCGCCGATGA	Nocardia cyriacigeorgica strain 3012STDY6756504 genome assembly, chromosome: 1	93.5
CAGGCCGCGGTGAGCGGTTCGGCCAACGACGCCGTCGCCGCCCGCGATGT	Nocardia cyriacigeorgica strain MDA3349 chromosome, complete genome	93.5
GAACCGATCCGAGATGAGGAGCCAATGAGCTACAACCCCTATGACGCTCT	Nocardia cyriacigeorgica strain MDA3349 chromosome, complete genome	93.5
CGGACGTTGTTGATCGTGAATCCGAATGCCACCTCTACCACCGCGGCCCC	Nocardia cyriacigeorgica strain 3012STDY6756504 genome assembly, chromosome: 1	89.8
GGTTTCCGCCCGGCCGAGGTCATCGCGCATGCAGCCGAATCTATTGCCCC	Nocardia cyriacigeorgica strain 3012STDY6756504 genome assembly, chromosome: 1	75.0
GCGGTCGATCGTGGCGACCGCGCCGTTATCGGCCGACGAGGACGACGAAC	Nocardia cyriacigeorgica strain NBC_00369 chromosome, complete genome	93.5
ATCCACAACCTACTCTTCGGTAGGACTATCCGAGTTCGGCGTCTGTTGGC	Nocardia cyriacigeorgica strain 3012STDY6756504 genome assembly, chromosome: 1	93.5
CTTTCACCTGCGCTGGTTCACCCCGCGGGTGGAGGTGGACATGTGCGGGC	Nocardia cyriacigeorgica strain 3012STDY6756504 genome assembly, chromosome: 1	93.5
GTGCAGCACGGCGAACAGGGCGACGTAATCCGAGCGCGGCACGCCCTGCG	Nocardia cyriacigeorgica strain 3012STDY6756504 genome assembly, chromosome: 1	93.5
ACCAGGCCTGCAGGATGTGGTTGTGCACGCTCCACCAGCCGAGATCGCGG	Nocardia cyriacigeorgica strain 3012STDY6756504 genome assembly, chromosome: 1	82.4

## Data Availability

No new data were created.
